# Vaccination against *Clostridium difficile* using toxin fragments: Observations and analysis in animal models

**DOI:** 10.4161/gmic.27712

**Published:** 2014-01-22

**Authors:** Janice Spencer, Rosanna Leuzzi, Anthony Buckley, June Irvine, Denise Candlish, Maria Scarselli, Gillian R Douce

**Affiliations:** 1 Institute of Infection, Immunity, and Inflammation; College of Medicine, Veterinary and Life Sciences; University of Glasgow; Glasgow, UK; 2 Novartis Vaccines and Diagnostics; Siena, Italy

**Keywords:** Clostridium difficile, vaccination, toxin fragments, neutralizing antibodies, hamster models, diarrhea, colonization factors, glucosyltransferase activity, protection

## Abstract

*Clostridium difficile* is a major cause of antibiotic associated diarrhea. Recently, we have shown that effective protection can be mediated in hamsters through the inclusion of specific recombinant fragments from toxin A and B in a systemically delivered vaccine. Interestingly while neutralizing antibodies to the binding domains of both toxin A and B are moderately protective, enhanced survival is observed when fragments from the glucosyltransferase region of toxin B replace those from the binding domain of this toxin. In this addendum, we discuss additional information that has been derived from such vaccination studies. This includes observations on efficacy and cross-protection against different ribotypes mediated by these vaccines and the challenges that remain for a vaccine which prevents clinical symptoms but not colonization. The use and value of vaccination both in the prevention of infection and for treatment of disease relapse will be discussed.

## Introduction

*Clostridium difficile* is a leading cause of antibiotic associated diarrhea and susceptibility to this infection increases with age, immunodeficiency, and antibiotic treatment^1^. While carriage of the organism within the gut can be asymptomatic, modification of the flora through antibiotic use frequently initiates disease. Symptoms ranging from mild to severe diarrhea are largely associated with the production of two large glucosyltransferase exotoxins, TcdA and TcdB^2^, which modify and damage the cellular architecture of the epithelial surface of the colon. This damage not only limits absorption of water but also induces through inflammasome activation a prolific inflammatory response including an influx of high numbers of polymorphonucleocytes (PMNs). While symptoms can be alleviated through the destruction and removal of the toxin-producing bacteria through treatment with metronidazole or vancomycin, further complications including pseudomembranous colitis, toxic megacolon, and sepsis also occur in a small number of cases^3^.

## Animal Models

The efficacy of any new treatment for *C. difficile* requires early evaluation in appropriate animal models. Until recently, the “gold standard” model for *C. difficile* infection was considered to be the Syrian Golden hamster. Unlike mice, the challenge of clindamycin treated animals with spores or vegetative cells results in an acute and fatal outcome with many symptoms including diarrhea and inflammation of the colon (including damage resembling pseudomembranous eruptions) similar to those observed in man. In contrast, genetically normal mice only appear to be transiently colonized when experimentally infected, with little or no changes in tissue pathology observed. However, work by Chen^4^ demonstrated that pre-treatment of mice with a combination of antibiotics in the drinking water modifies the microbiota such that subsequent exposure to clindamycin and *C. difficile* spores results in significant tissue pathology and in some cases death. Disease severity can be measured indirectly by monitoring mouse weight, which drops significantly 2 days post infection. This weight loss appears transient with recovery to normal weight observed 4 days post infection. A less severe but useful mouse model is the “transmission model” which models spread of this infection between susceptible individuals. In this model, mice infected with *C. difficile* transiently shed high levels of the organism within the feces. Approximately 1 week post challenge, infection appears cleared as recovery of detectable bacteria in the feces is difficult. However, the organism appears to be retained at low levels as subsequent treatment with clindamycin and/or vancomycin provokes the outgrowth, with *C. difficile* being readily detected in the faces. Under certain circumstances, some animals develop “supershedder” status, continually secreting high levels of spores within the feces. This model has been very elegantly used to evaluate the role of the microbiota in disease and the fitness of particular ribotypes to dominate this niche in vivo^5^.

*C. difficile* infects a wide range of animals and is one of the major causes of enteritis in neonatal pigs. Oral infection of such pigs experimentally with toxin-producing strains results in severe inflammation within the large bowel. Interestingly modification of the dose and age of the pig can influence both the severity (from acute and severe disease) and type (mild vs. chronic) disease^6^. While housing and cost has limited widespread use of the model, the recently discovered overlap in strains recovered from man and pigs suggests that these animals may provide the ultimate model for this disease. If pigs provide a reservoir of infection for this pathogen, then development of a swine vaccine may be appropriate as we look to reduce and/or eliminate the bacterial burden of infection within the environment.

## Vaccination to Prevent *C. difficile*

While current treatments for symptomatic disease are based upon the administration of additional antibiotics, frequent recurrence of disease following withdrawal of treatment has strengthened the need for alternative approaches. Vaccination using toxoids or recombinant fragments has been tested in both animals and man with varying and limited success. Protection appears dependent on the production of high levels of neutralizing antibody to both toxin A and toxin B. In fact, passive transfer of monoclonal antibodies to these toxins provides protection against *C. difficile* in human subjects^7^. In man, the presence of high levels of systemic toxin A antibodies alone appears to correlate with protection from *Clostridium difficile* associated diarrhea (CDAD) ^8^. In contrast, there is some evidence that the level of neutralizing antibodies to toxin B correlates with the prevention of disease and relapse^9^^,^^10^. While protection has largely been attributed to neutralizing antibodies generated to the binding domains of these toxins, recent work by this group and confirmed by others^11^ has indicated that high titers of neutralizing antibodies to toxin B can be generated using regions other than the binding domain. These antibodies appear to provide greater protection in animal models as well as reducing the longevity and severity of diarrheal symptoms associated with this disease.

## Protective Efficacy of Toxin Repetitive Binding Domains (RBD)

Early studies in animals and more recent clinical studies in man have focused on the use of detoxified versions of the proteins to generate protective immunity^12^^-^^16^. A summary of such vaccines is given in Table 1. However, difficulties in the manufacture and efficacy of the toxoid based vaccines including variation in the quantity and quality of neutralizing antibodies generated suggest an opportunity for improvement. Several groups have considered the use of recombinant antigens based on the repetitive binding domains (RBD) located at the C terminus of both toxins^17^^-^^19^. X-ray structural analysis of this region in TcdA revealed that these sequences fold into repetitive solenoid-like structures^20^ that have potential as vaccine candidates. This was first demonstrated in the 1990s by Lyerly, who showed that a recombinant protein encoding 33 of the 38 regions of toxin A were sufficient to protect animals against toxin A challenge^21^. These fragments generated neutralizing antibodies which appeared essential in prevention of lethality in the Syrian Golden hamster model of infection. Over the intervening years, several formulations of experimental vaccines have been tested using different routes of immunisation and delivery vehicles to enhance immune responses^22^^-^^25^ (Table 1). More recently, groups^18^^,^^19^ have revised the formulation to include toxin B, as fragments of toxin A alone failed to provide full protection. This has shown that systemic vaccination of RBDs of both toxins can prevent lethal disease.

**Table:** **Table 1.** Summary of vaccines formulations against *C. difficile* described in the literature

Toxoids preparations	Animal model/clinical study	Route of immunization	References
Toxoid A and B from culture filtrates	Hamster	Parenteral (i.p., s.c.) + mucosal (i.n., i.g., r.)	13
Partially Purified toxoid A and B	Hamster	Parenteral (i.m.) + mucosal (i.n., i.g., r.)	12
Partially Purified toxoid A and B	Healthy adults	4 doses i.m.	14, 44
Partially Purified toxoid A and B Purified toxoid B	Patients Hamster	4 doses i.m 3 doses i.p	45 16
Highly Purified toxoid A and B (> 90%)	Hamster	i.m.	46
Highly Purified toxoid A and B (> 90%)	Healthy adults, elderly	3 doses i.m	15
Genetically modified toxoid A and B	Hamster	i.m.	47

Recombinant toxin-based antigens			
RBD TcdA	Hamster	subcutaneous	21
RBD TcdA	Rabbit	Oral/*V. cholerae* vector	22
RBD TcdA	Mice	i.n., i.g.	17
RBD TcdA/B	Mice, hamster	i.g./*B. subtilis* spores	24
RBD TcdA	Mice	DNA vaccine, i.m.	23
RBD TcdA	Mice	adenovirus vaccine, i.m.	25
Fusion protein RBDs of TcdA/B	Mice, hamster, monkey	i.m.	18
ToxinA/B chimeric protein	Mice, hamster	i.m., i.p.	11
RBDs and GT of TcdA/B	Mice, hamster	i.p.	19

Surface antigens			
Crude SLP	Mice, hamster	i.p., i.n.	34
FliD	Mice	i.n., r., i.g./ PLGA encapsulation	35
Cwp84	Hamster	s.c., r., i.g	36
Cwp84	Hamster	i.g./ pectin beads encapsulation	37

i.p., intraperitoneal; s.c., subcutaneous; i.n., intranasal; i.g., intragastric; r., rectal; i.m., intramuscular

Most of the RBD fragments used in such studies have been cloned using genomic sequences from two strains of *C. difficile*, the highly toxic strain VPI10463 or 630 from which the first annotated sequence was generated^26^.While these fragments generate strong neutralizing antibodies, documented variation in the toxin B RBD may limit the potential of these antigens to completely neutralize the activity of variant toxins. Using RBD fragments of toxin A (2387–2710 nt) and toxin B (1853–2366 nt) cloned from 630, we have shown varying degrees of protection in the hamster model using 3 different strains of *C. difficile* (Fig. 1). These differences may reflect either variation in the neutralizing capacity of the antibody to divergent toxins or differences in the amount and activity of the toxins produced in vivo. In our hands and confirmed in observations by others^27^ 630 appears to generate less toxin over an equivalent time period than *C. difficile* strains B1 or R20291.

**Figure:**
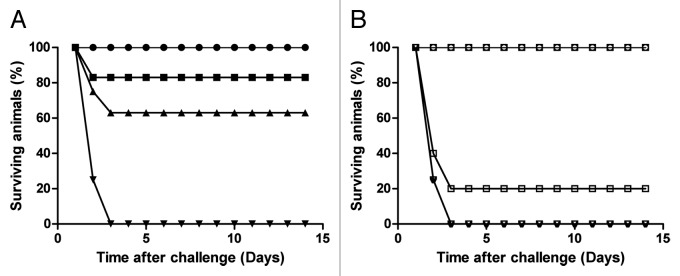
**Figure 1.** Survival of clindamycin treated vaccinated hamsters challenged with *C. difficile*. (**A**) shows the survival of animals following vaccination with RBD-TcdA_630_ and RBD-TcdB_630_ (50 μg per dose and/or 4 vaccinations) and challenged with *C. difficile* 630 (closed circles), B1 (closed square), and R20291 (closed triangles). Unvaccinated controls for each strain are also included (closed diamonds). (**B**) shows the survival time of animals vaccinated with 4 doses of either RBD-TcdA_630_ and RBD-TcdB_630_ (open squares) or RBD-TcdA_630_ and TcdB-GT_630_ open circles) 30 μg per dose and challenged with *C. difficile* B1. Unvaccinated controls for each strain are also included (open triangles). Each experiment represents a minimum of 6 animals per group. Differences in survival for animals immunized with RBD-TcdA630 and RBD-TcdB_630_ between (**A**) and (**B**) may reflect the impact of a lower dose of proteins given to animals challenged with *C. difficile* B1 in (**B**).

Variation in the amount of antigen used in vaccination has been shown to influence the level of protection with animals vaccinated with a reduced formulation (30 μg per dose of RBDs from TcdA and TcdB) being more susceptible to fatal disease (Fig. 1B). This appears particularly true for the RBD from toxin B, which in general seems to be less immunogenic than the equivalent toxin A protein. Whilst high titers of antibodies to toxin A have been observed after single vaccinations, several vaccinations are required to generate even the most limited anti toxin B response^13^.

## Identification of Alternative and Effective Neutralising Epitopes for Toxin B

In the last decade the number and complexity of *C. difficile* cases have increased worldwide and while a proportion of these cases can be attributed to increased vigilance, the emergence and spread globally of a number of hypervirulent and epidemic strains has also contributed. In the UK, the progenitor of the epidemic 027 ribotype (known as R20291 or UK1) was isolated in 2006 at Stoke Mandeville during an outbreak that resulted in over 30 deaths. The spread and evolution of this strain worldwide has recently been documented^28^, although a conclusive explanation for its rapid spread and dominance remains elusive. However, it is clear that vaccines of the future need to show clear efficacy against such strains. While the use of a TcdB toxoid appears to generate immunity that is cross protective between phylogenetically distinct *C. difficile* strains^16^ , the RBD region of this toxin has been reported to be variable between different toxinotypes^2^^,^^29^^,^^30^. We and others^11^ have considered the use of other more conserved regions of the toxin as vaccine candidates. More specifically, we have shown that a recombinant fragment encoding the glucosyltransferase activity of toxin B (TcdB-GT 1–543 nt) in combination with the RBD region of toxin A can raise neutralizing antibodies. This fragment appears to generate superior protective responses to the equivalent RBD of toxin B when used in combination with RBD from toxin A in parallel experiments (Fig. 1B). Inclusion of this region has also been shown in the mouse model of relapsing disease to reduce recurrence of the disease. This result provides an argument for inclusion of these domains in future superior vaccines. The potential of the catalytic domain as a source of toxin B neutralizing epitopes has further been confirmed through the creation of a chimeric protein in which the RBD of toxin B was exchanged for the equivalent RBD from toxin A^11^.

## Protection from Local or Systemic Toxic Activity

While activity of the toxin at the mucosal barrier is well documented, the systemic impact of toxin action is less clear. Hamsters, unlike mice, appear acutely sensitive to the toxins, with animals succumbing to an acute lethal disease between 24–48 h following oral challenge. These animals do not appear to die as a consequence of dehydration attributed to diarrhea as animals that show intermediate levels of protection suffer several episodes of diarrhea followed by recovery. In contrast, naive infected animals develop diarrhea and rapidly become moribund. Data from zebrafish^31^, and more recently from gnotobiotic piglets^10^, have highlighted the sensitivity of cardio and pulmonary tissue to *C. difficile* toxins. While no gross pathology has been observed in these tissues in hamsters, our current use of biotelemetry has indicated a role for these toxins systemically. Animals with acute infection show a short rise in body temperature followed by a rapid and sustained reduction that is associated with organ failure (Fig. 2). In contrast, vaccinated animals show a small but sustained elevation at the same time as the naive animals before a return to normal biorhythms. This elevation in temperature may reflect the release of cytokines such as interleukin 1β (IL-1β) ^32^, which have been shown to be released from macrophages exposed to either TcdA or TcdB. This cytokine together with IL-6 has been shown to be higher in pigs and mice with systemic CDI and may be responsible for the increase in body temperature observed^33^. Neutralization of toxins as a consequence of vaccination may limit inflammatory cytokine release and alter the subsequent downstream effects which in the hamsters are lethal. At present, it is unclear whether systemic toxicity has a role clinically, and it will be difficult to determine given that the majority of infections occur in elderly hospitalized patients with complicated medical histories. However, it may be speculated that these toxins contribute indirectly to organ failures in these weakened patients. These observations further support the use and development of vaccines against the toxins.

**Figure:**
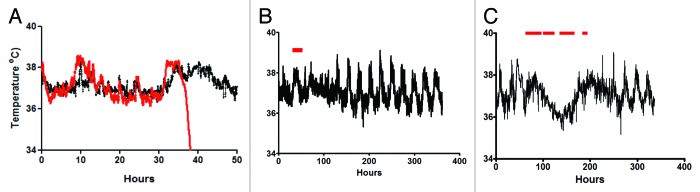
**Figure 2.** Body temperature fluctuations in naive and vaccinated hamsters infected with *C. difficile*. (**A**) represents the typical change in temperature observed in a naive animal (red line) and a vaccinated animal (black line) during the same stage of infection. Readings are taken every minute from an implanted chip within the infected animal. (**B**) represents the typical profile of temperature variation in a vaccinated and protected animal during a 14 day experiment. The red bar represents the time over which diarrhea was observed. (**C**) represents the profile observed in partially protected animals, which display intermittent episodes of diarrhea and recovery.

## Protection from Toxin Mediated Symptoms but Not Colonization

Systemic vaccination with *C. difficile* toxoid vaccines have been shown to generate high levels of serum antibodies that appear to reduce the potential for relapse. The difficulty with this approach is the availability of strong toxin-neutralizing activity at the epithelial barrier. In systemically vaccinated and protected animals, short and self-limiting episodes of diarrhea are frequently observed^11^^,^^14^. It has been suggested that this reflects damage to the gut epithelium, initiated by early toxin production, which releases toxin neutralizing antibodies from the local vasculature of the gut. Early studies, in which mucosal vs. systemic vaccinations were compared, indicated that only the production of mucosal responses through a combination of systemic and mucosal immunization limited these symptoms^13^. In our hands mucosal vaccination alone with recombinant fragments of receptor binding domains elongates survival time (by approx 10 h) but does not prevent the eventual systemic impact of the toxin (data not shown). This suggests that strategies that activate both mucosal and systemic responses would be optimal for complete prevention of symptoms.

However, is prevention of symptoms sufficient? In our hands, vaccinated animals that show limited or no diarrheal symptoms continue to shed the organism at high levels in the feces for up to 3 weeks post infection (Fig. 3A). This would suggest that while vaccination prevents toxin mediated symptoms it is not sufficient to prevent outgrowth, sporulation, and release of the spores into the environment. As current clinical diagnosis is dependent upon detection of toxins in fecal samples, it is possible that a vaccination strategy designed to prevent clinical symptoms could lead to an underestimation of the extent of colonization in a given target population. An ideal vaccine therefore should additionally be formulated to include bacterial factors that also limit colonization. One complication of this approach is the lack of information as to which bacterial proteins contribute to epithelial adhesion and long-term persistence. Several surface exposed antigens have been proposed including SLP, FliD, and cwp84 (Table 1) ^34^^-^^37^. The impact of inclusion of these antigens in vaccine formulations has been found to vary levels of colonization. A further complication may be the location and nature of the bacterial interaction with the epithelial barrier. Evidence produced by electron microscopy and immunofluorescence suggest that while aggregates of bacteria appear associated with the epithelial barrier (Fig. 3B) bacterial counts (both vegetative and spores) recovered from the cecum and colon are in significantly higher numbers than imaging of the tissue would suggest. As a consequence it is difficult to determine whether attachment to the epithelial barrier is an essential requirement for toxin production and disease sequelae. While the usefulness of anti-colonization factors in acute disease is unclear, the potential to eradicate low grade persistent infection is attractive. In our studies, animals in the acute stage of infection show high levels of sporulation with spores detectable in both the feces and directly from gut samples (approximately 90% of the organisms recovered). In contrast, 14 days post infection of vaccinated animals or animals infected with naturally toxin deficient strains of the organism, show a much lower proportion of the recovered bacteria as spores (less than 50%) (Fig. 3A). In these animals, the majority of recovered organisms are vegetative cells and this may indicate that the organism is retained and can continue to persist as a member of the normal microbiome. The ability of *C. difficile* to generate biofilms in vitro^38^^,^^39^ may also play a role in protection from subsequent antibiotic treatment and this could be prevented if initial colonization was limited.

**Figure:**
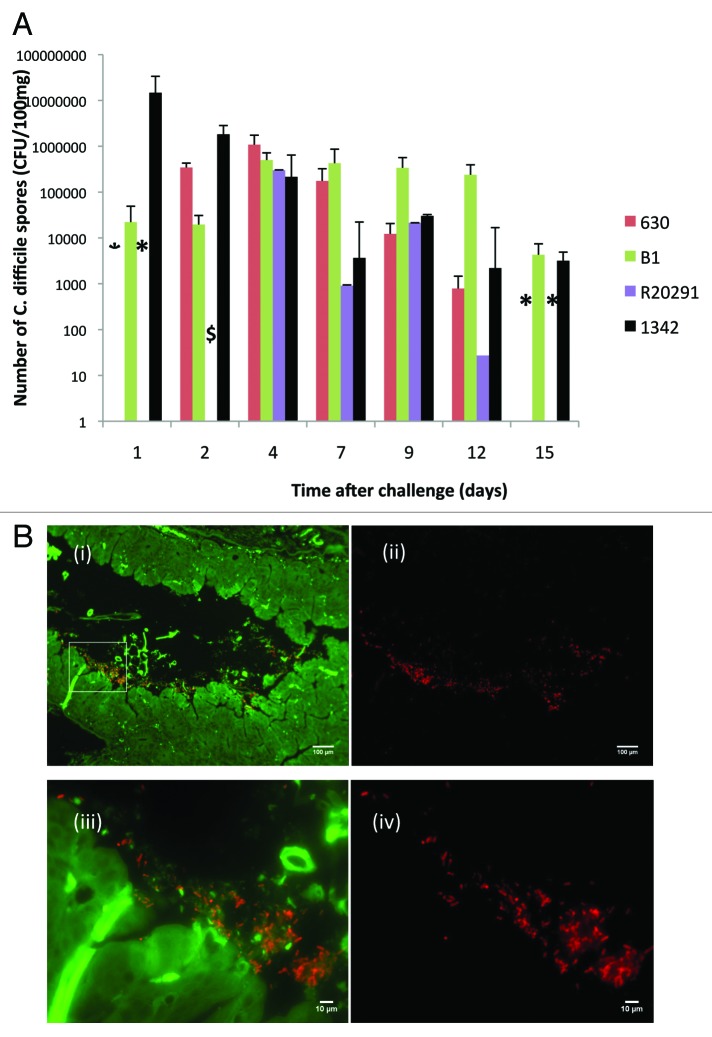
**Figure 3.** Bacteria in the feces in vaccinated animals challenged with three toxic strains (630, B1, or R20291) or naive animals infected with naturally non-toxic strain of *C. difficile*. (**A**) shows the recovery of *C. difficile* (cfu per 100 mg feces) over time in vaccinated animals following challenge with strains 630 (red), B1 (green), R20291 (purple), and naive animals infected with paLoc negative strain 1342 (black). Error bars represent the standard error of the mean cfu counts from a minimum of 6 animals per strain. * indicates time points at which spores were not recovered and ^$^ time point at which no feces were recoverable due to diarrheal episode (**B**). Immunofluorescence of paLoc negative strain 1342 in cecal tissue 1 day post infection. 3b (i) shows low power magnification of infected tissue in which *C. difficile* are stained with anti-surface layer protein A (slpA) antibodies. Images are observed at 490 nm (green fluorescent protein, gfp highlighting tissue) and 580nm (red fluorescent protein (rfp), highlighting *C. difficile*). 3b (ii) is identical image from (i) using the 580nm filter alone. 3b (iii) shows magnification of highlighted region from 3b (i) using 490 and 580 filter sets. 3b (iv) is the image from (iii) using the 580nm filter alone. Images were prepared from formaldehyde fixed tissue. De-waxed sections were stained with anti-rabbit slpA antibody (1:1000; 90 min; 37 °C) and counterstained with goat anti rabbit IgG conjugated with Alexa Fluor 555 (Life Technologies; 1:1000; 90 min; 37 °C). Stained slides were visualized using a Zeiss Imager M1 microscope with images taken using a Hamamastsu ORCA-ER digital camera and Improvision acquisition Hub. Images were analyzed using Volocity 3D image analysis software version 5.5 (Perkin Elmer Inc).

## Reducing Clinical Disease and Environmental Contamination

One of the most clinically challenging problems of *C. difficile* infection is the treatment of patients who initially respond to first line antibiotics (vancomycin and metronidazole) but suffer a subsequent recurrence of symptoms when these drugs are withdrawn^40^. Evidence suggests that patients who suffer a relapse either as a result of reactivation of a pre-existing infection, or infection with a different strain, are more likely to suffer subsequent episodes of the disease.^41^ Susceptibility to relapse does appear to correlate with long-term modification of the gut microbiota, with microbial diversity a key factor in the control of outgrowth and subsequent toxin expression and release. This is most apparent in the elderly who suffer higher incidences of infection than younger patients exposed to equivalent antibiotic treatment. Further studies within the elderly in Ireland have recently shown that hospitalized patients show a much reduced diversity compared with healthy age matched individuals living within their own homes^42^. At this stage it is not clear whether bacteriostatic products produced by members of the microbiome or modification to host proteins, including those involved in epithelial barrier integrity and immunity, play a role in controlling C. *difficile* outgrowth. Combining vaccination with long-term modifications to the microbiota through the use of bacteriotherapy could serve to both limit those with disease and long-term contamination of the environment.

## Conclusion and Further Questions for Development

Vaccination with recombinant fragments from toxin A and toxin B can protect hamsters against lethal challenge with *C. difficile.* While the RBD of toxin A has been shown in several studies to generate strong neutralizing and protective antibodies, less is known about the most effective antigen from toxin B. We and others have proposed that fragments encoding the glucosyltransferase activity of toxin B provide a vaccine candidate that is conserved and that generates strong neutralizing activity. The combination of these two fragments generates superior protection to that observed when the RBD region of toxin B is included in the formulation. As a consequence, we propose that an optimal vaccine against *C. difficile* would include this fragment. Future improvements for a vaccine formulation should also include the identification of anti-colonization factors to limit long-term survival of the organism within the host. This has implications for reduction in the rates of relapse and may help to reduce environmental contamination^43^. While vaccination of the elderly can be difficult, experience with both pneumococcal and influenza vaccines have shown this approach has value. Identification of strong vaccine candidates that have the potential, even in this current form, to eliminate or even reduce the debilitating and distressing diarrheal symptoms associated with this disease is attractive. Its use in combination with other antigens or with other therapies provides hope in the longer term for reduction of environmental contamination and source of infection in our hospitals and health care institutions.

## Notes

LeuzziRSpencerJBuckleyABrettoniCMartinelliMTulliLMarchiSLuzziEIrvineJCandlishD Protective efficacy induced by recombinant Clostridium difficile toxin fragments Infect Immun 2013 81 2851 60 10.1128/IAI.01341-12

## Notes

10.4161/gmic.27712
